# Neuroligin 1, 2, and 3 Regulation at the Synapse: FMRP-Dependent Translation and Activity-Induced Proteolytic Cleavage

**DOI:** 10.1007/s12035-018-1243-1

**Published:** 2018-07-28

**Authors:** Joanna J. Chmielewska, Bozena Kuzniewska, Jacek Milek, Katarzyna Urbanska, Magdalena Dziembowska

**Affiliations:** 1 Laboratory of Molecular Basis of Synaptic Plasticity, Centre of New Technologies, University of Warsaw, S. Banacha 2c, 02-097 Warsaw, Poland; 2 Postgraduate School of Molecular Medicine, Medical University of Warsaw, Zwirki i Wigury 61, 02-091 Warsaw, Poland

**Keywords:** Neuroligins, FMRP, Local translation, Synapse, Fragile X syndrome, Proteolysis

## Abstract

Neuroligins (NLGNs) are cell adhesion molecules located on the postsynaptic side of the synapse that interact with their presynaptic partners neurexins to maintain trans-synaptic connection. *Fragile X* syndrome (FXS) is a common neurodevelopmental disease that often co-occurs with autism and is caused by the lack of fragile X mental retardation protein (FMRP) expression. To gain an insight into the molecular interactions between the autism-related genes, we sought to determine whether FMRP controls the synaptic levels of NLGNs. We show evidences that FMRP associates with *Nlgn1*, *Nlgn2*, and *Nlgn3* mRNAs in vitro in both synaptoneurosomes and neuronal cultures. Next, we confirm local translation of *Nlgn1*, *Nlgn2*, and *Nlgn3* mRNAs to be synaptically regulated by FMRP. As a consequence of elevated *Nlgns* mRNA translation *Fmr1* KO mice exhibit increased incorporation of NLGN1 and NLGN3 into the postsynaptic membrane. Finally, we show that neuroligins synaptic level is precisely and dynamically regulated by their rapid proteolytic cleavage upon NMDA receptor stimulation in both wild type and *Fmr1* KO mice. In aggregate, our study provides a novel approach to understand the molecular basis of FXS by linking the dysregulated synaptic expression of NLGNs with FMRP.

**Electronic supplementary material:**

The online version of this article (10.1007/s12035-018-1243-1) contains supplementary material, which is available to authorized users.

## Introduction

Neuroligins (NLGNs) are cell adhesion molecules located on the postsynaptic side of the synapse that interact with their presynaptic partners neurexins to maintain trans-synaptic connection [1–3]. Three highly homologous neuroligins are abundantly expressed in rodent brain: NLGN1, NLGN2, NLGN3 [4], while NLGN4 is less abundant in rodent brain and less evolutionarily conserved among all NLGNs isoforms [5, 6]. Despite their co-expression in the same neuron, distinct neuroligins exert specific functions depending on the type of the synapse [7]. NLGN1 is present predominantly at excitatory synapses [8, 9], NLGN2 and NLGN4 localize at inhibitory synapses [9–13], while NLGN3 is found on both subsets of synapses [14, 15]. Neuroligins are delivered by a vesicular transport to the postsynaptic membrane, where they function as homo- or heterodimers [15]. One of the first genetic mutations associated with autism were found in neuroligin 3 and 4 genes [16, 17]. The subsequent studies in genetically modified mice revealed that the discovered mutations interfere with neuroligin trafficking to the postsynaptic membrane, by enhancing their intracellular retention and thus diminishing their synaptic level [18–21]. Although it was initially thought that neuroligins are indispensable for synapse formation, research on NLGN1/2/3 triple KO mice has revealed that they are predominantly required for proper synapse maturation and brain function [6]. Many recent studies indicate that the role of neuroligins goes far beyond the simple scaffold formation, for example neuroligins can regulate such intrinsic synaptic functions as synaptic levels of NMDA (*N*-methyl-D-aspartate) and AMPA (α-amino-3-hydroxy-5-methyl-4-isoxazolepropionic acid) receptors [1, 19, 21–24]. It has also been shown that nascent neurexin-neuroligin contacts rapidly capture surface-diffusing AMPARs at PSD-95 (postsynaptic density protein 95) scaffolds [25]. At inhibitory synapses, C-termini of NLGN2 couple to collybistin and gephyrin and influence GABA_A_ (γ-aminobutyric acid) receptor function [11, 26].

*Fragile X* syndrome (FXS) is the common neurodevelopmental disease that often co-occurs with autism spectrum disorder (ASD) and is then described as syndromic autism [27]. FXS results from CGG repeat expansion in the 5′ UTR of *FMR1* gene, which causes *FMR1* gene silencing and the absence of fragile X mental retardation protein (FMRP) [28]. FMRP is an RNA-binding protein highly expressed in neurons that has been shown to bind to a number of synaptic mRNAs and temporarily inhibit their translation by stalling the ribosomal translocation [29]. In response to neuronal stimulation, FMRP is dephosphorylated and dissociates from the polyribosomes thus enabling the protein synthesis [30–32]. FMRP regulates translation of many transcripts involved in neuronal maturation, proper synaptic signaling, and plasticity [29, 33–36]. The lack of FMRP-dependent translational repression in FXS leads to dysregulated local synthesis of synaptic proteins what results in impaired synaptic functions and patient’s cognitive impairment [28, 37].

To gain an insight into the molecular interactions between the ASD-related genes, we sought to determine whether FMRP controls the synaptic level of NLGNs. We show evidence that FMRP associates with *Nlgn1*, *Nlgn2*, and *Nlgn3* mRNAs in immunoprecipitation assay with anti-FMRP antibody*.* Next, we prove that *Nlgn1*, *Nlgn2*, and *Nlgn3* mRNAs local translation at the synapse is dysregulated in *Fmr1* KO mice, the model of FXS. As a consequence of elevated *Nlgns* mRNA translation *Fmr1* KO mice exhibit increased incorporation of NLGN1 and NLGN3 into the postsynaptic membrane. Finally, we show that synaptic levels of all neuroligins are precisely and dynamically regulated by their rapid proteolytic cleavage upon NMDA receptor stimulation in both wild type and *Fmr1* knockouts.

## Materials and Methods

### Animals

We used male *Fmr1* knockout (*Fmr1* KO) mice on FVB background (FVB.129-Fmr1^tm1Rbd^/J, RRID:IMSR_JAX:008909) and their wild-type (WT) littermates. The age of animals used in each experiment is indicated in the description of the experiment. Prior to the experiment, the animals were kept in the animal facility with ad libitum access to food and water with a 12-h light/dark cycle. The mice were genotyped accordingly to the Jackson Laboratory protocol. The mice were handled in accordance with the ethical standards of European and Polish regulations. *Nlgn3* knockout (*Nlgn3* KO) mice used as a control for anti-NLGN3 antibody specificity were a kind gift from Prof. Nils Brose.

### Synaptoneurosomes Preparation and NMDAR Stimulation

Synaptoneurosomes were prepared from male WT and *Fmr1* KO mice by differential filtration as described previously by us and others [38–40]. Briefly, hippocampi and a part of adjacent cerebral cortex (to maintain cortico-hippocampal synaptic connections) from one mouse was dissected and homogenized in 1.5 ml of ice-cold homogenization buffer (HB) containing (in mM) 118.5 NaCl, 1.18 MgSO_4_, 1.18 KH_2_PO_4_, 2.5 CaCl_2_, 3.8 MgCl_2_, 212.7 glucose, 24.9 NaHCO_3_, pH 7.4 set with carbogen, supplemented with protease inhibitor cocktail cOmplete EDTA-free (Roche), and, for RNA isolation, 120 U/ml of RiboLock RNase Inhibitor (Thermo Scientific, Waltham, MA). The homogenate was resuspended in 10 ml of HB per one brain, filtrated through a series of nylon mesh filters (Merck Millipore, Kenilworth, NJ): 100, 60, 30, and 10 μm consecutively, and centrifuged at 1000 rcf for 15 min at 4 °C. The pellets containing synaptoneurosome preparations were washed once in 5 ml of HB, centrifuged as before and resuspended in 1 ml of HB. The purity of synaptoneurosomal preparations was verified as described before [38, 40]. The protocol for in vitro stimulation of NMDA receptors (NMDAR) on synaptoneurosomes was described before [38, 41, 42]. The aliquots of freshly isolated synaptoneurosomes were prewarmed for 3 min at 37 °C and then NMDA (50 μM final conc.) and glutamate (10 μM final conc.; Sigma-Aldrich, St. Louis, MO) were added for 30 s followed by immediate APV (DL-2-Amino-5-phosphonovaleric acid, Sigma-Aldrich) application (120 μM final conc.). The incubation was carried out for 2.5, 5, 10, and 20 min at 37 °C with 1000 rpm shaking. The unstimulated samples (basal state, Bsl) were left on ice. In Fig. 7d–f, male P30-40 WT synaptoneurosomes were incubated with MMP-9/-13 inhibitor I (5 μm final conc.; #444252, Calbiochem) for 10 min on ice before NMDAR stimulation and incubation for 2.5 min. Male P30-40 WT and *Fmr1* KO mice were used for isolation of synaptoneurosomes in Fig. 3a–c, g. Male P30-40 WT and *Fmr1* KO mice were used to show activity-dependent NLGNs cleavage in Fig. 7a–c, while male P40 WT and *Nlgn3* KO mice were used in Fig. 7g.

### RNA Co-immunoprecipitation and qRT-PCR

The RNA co-immunoprecipitation with anti-FMRP antibody was performed as published before [40, 43]. Briefly, 1.6 mg of total protein from freshly prepared synaptoneurosomes from hippocampi and a part of adjacent cerebral cortex of male P30-40 WT and *Fmr1* KO mice were lysed in 1200 μl precipitation buffer (10 mM HEPES, pH 7.4, 200 mM NaCl, 30 mM EDTA, 0.5% Triton X-100) with protease inhibitor cocktail cOmplete (Roche, Indianapolis, IN) and 80 U/ml RiboLock RNase Inhibitor (Thermo Scientific). The protein concentration was measured by Bradford protein assay (Bio-Rad, Hercules, CA). The preclearing was performed with 3.6 mg of washed Dynabeads Protein A (Invitrogen, Carlsbad, CA) for 4 h. Afterwards, 100 μl of each supernatant was taken as an input fraction for western blot with anti-FMRP antibody to check the procedure efficiency. The precleared samples were precipitated overnight in 4 °C while rotating with 120 μl of antibody-bound Dynabeads Protein A, with either anti-FMRP antibody (7G1-1, Developmental Studies Hybridoma Bank, Iowa City, IA) or normal mouse IgG (#sc-2025, Santa Cruz Biotechnology, Dallas, TX). Then, 1/5 of the Dynabeads was washed and boiled in Laemmli buffer containing 100 mM DTT for western blot and total RNA was extracted with TRIzol Reagent (Ambion, Austin, TX) from the remaining 4/5 of the washed Dynabeads. Concentration and quality of RNA was checked using DS-11 Spectrophotometer (DeNovix, Wilmington, DE). After reverse transcription using random primers (GeneON, #S300, Ludwigshafen am Rhein, Germany; 200 ng per reaction) and SuperScript IV Reverse Transcriptase (Thermo Scientific), quantitative real-time (qRT)-PCR was performed using Light Cycler 480 Probes Master Mix (Roche) and TaqMan primer/probes: Mm00492193_m1 for *Psd95*, Mm99999915_g1 for *Gapdh*, Mm02344307_m1 for *Nlgn1*, Mm01703404_m1 for *Nlgn2* and Mm01225951_m1 for *Nlgn3* (Thermo Scientific). Calculations were performed using 2(-ddCt) method. Statistical analysis from three to four independent experiments (*n* = 3–4) was performed using one-way ANOVA followed by post hoc Sidak’s multiple comparisons test in GraphPad Prism version 7.03.

### Primary Neuronal Cultures

The culture of primary hippocampal neurons was set from P0 WT and *Fmr1* KO mice as well as P0 WT Wistar rats of either sex. About 120,000 dissociated neurons were plated on coverslips coated with 50 μg/ml poly-D-lysine (Sigma-Aldrich) and 2.5 μg/ml laminin **(**Roche**)** in 12-well plastic culture plates. The medium consisting of Neurobasal A (Gibco, Waltham, MA) supplemented with B-27 (Gibco), 1% penicillin-streptomycin (Sigma-Aldrich), 0.5 mM L-glutamine (Sigma-Aldrich), and 12.5 μM glutamate (Sigma-Aldrich) was replaced with fresh medium at DIV1 (in the case of murine cultures) and the cultures were developed in 5% CO_2_ incubator at 37 °C. Cross-linking and lysis (description below) was performed at DIV19, while lysis for basal state NLGN protein level measurements was performed at DIV21. The protein concentration before western blotting was measured using Pierce BCA protein assay (Thermo Scientific). The results of basal protein level in hippocampal cultures were quantified from *n* = 5 to 9 (Fig. 3d–f). At DIV21, the rat cultures were fixed with 4% paraformaldehyde/4% sucrose in PBS for FISH and immunostaining.

### Fluorescence In Situ Hybridization and Immunostaining

**Fig. 1:**
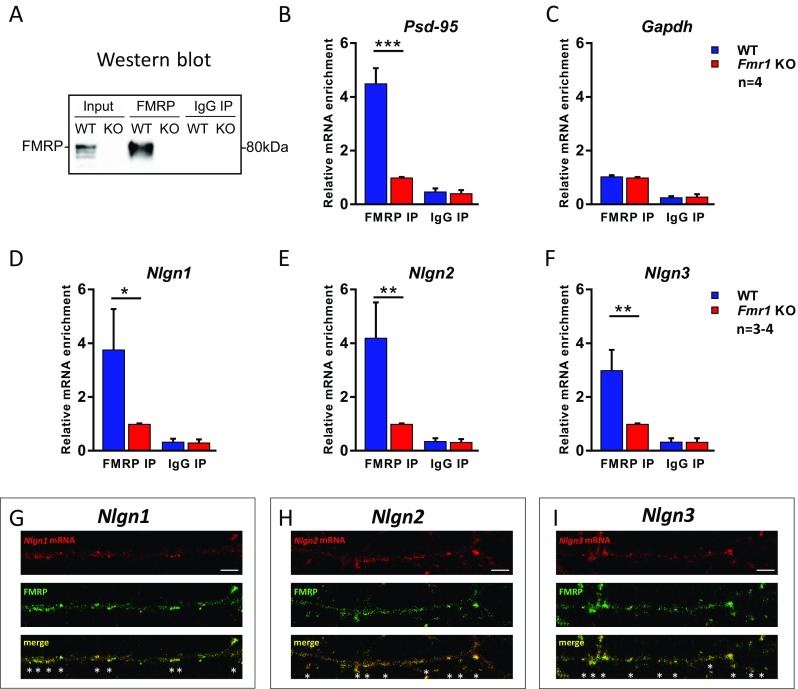
*Nlgn1*, *Nlgn2*, and *Nlgn3* mRNAs associate with FMRP in synaptoneurosomes and in cultured hippocampal neurons. **a** Western blot analysis of immunoprecipitated FMRP from mouse synaptoneurosomes shows FMRP in input and FMRP IP from WT mice. *Fmr1* KO extracts and IgG IPs were used as controls. **b**–**f** qRT-PCR analysis of mRNAs immunoprecipitated with anti-FMRP 7G1-1 antibody and separately with IgG for: **b** *Psd-95* (positive control), **c** *Gapdh* (negative control), **d** *Nlgn1*, **e** *Nlgn2*, **f** *Nlgn3*. The graphs show the enrichment of mRNAs detected in WT over *Fmr1* KO immunoprecipitates. Data represent mean values ± SEM, *n* = 3–4, *p < 0.05, **p < 0.01, ***p < 0.001 by one-way ANOVA followed by post hoc Sidak’s multiple comparisons test. **g**–**i** Fluorescence in situ hybridization with antisense riboprobes for **g** *Nlgn1*, **h** *Nlgn2*, and **i** *Nlgn3* mRNAs and immunofluorescence staining for FMRP shows partial colocalization of *Nlgns* mRNAs with FMRP in dendrites of DIV21 rat hippocampal neurons (asterisk). Scale bars, 5 μm

Antisense and sense (control) oligonucleotide probes were designed to the part of coding sequence and 3′ UTR of *Nlgn1*, *Nlgn2*, and *Nlgn3* rat cDNA for FISH. The sequences were amplified by PCR using the following primers:

- F: 5′CACTCGAACTTTGGCTCACC3′ R: 5′GGAAAGGCTGATGTGACTGG3′ for *Nlgn1*; F: 5′TCCGCCAGACACAGATATCC3′ R: 5′CCCAAAGGCAATGTGGTAGC3′ for *Nlgn2*; F: 5′AGCTCTACCTTCACATCGGG3′ R: 5′GACCCAACTGTAATGCTGCC3′ for *Nlgn3* and cloned into pCR II plasmid (TA Cloning Kit, Invitrogen). Linearized plasmids served as templates for in vitro transcription using the Fluorescein RNA Labeling Kit and SP6/T7 RNA Polymerases (Roche). Fluorescein-labeled probes were hybridized to fixed rat cultured neurons as described previously [40]. Briefly, the fixed hippocampal neurons were washed in PBS, treated with H_2_O_2_, and acetylated. The prehybridization solution with 50% formamide (Sigma-Aldrich) was applied for 3 h and replaced with hybridization solution (Sigma-Aldrich) with sense or antisense riboprobes for overnight incubation in 67.5 °C. Next, the cells were washed 3× in 0.5× saline-sodium citrate buffer (SSC) at 65 °C, 3× in 0.2× SSC at 60 °C, 2× in 0.2× SSC at room temperature (RT), and 3× in PBS-Tween 0.1% (PBST) at RT. Then, the unspecific binding was blocked with 10% NGS in TNB blocking solution (TSA Plus System; PerkinElmer) for 1 h at RT. The cells were incubated with anti-fluorescein-POD (1:200 in TNB, Roche) and rabbit anti-FMRP antibodies (1:200 in TNB, #7104S, Cell Signaling) in humidified chamber overnight in 4 °C, washed with PBST, and incubated with Alexa 488-conjugated anti-rabbit IgG secondary antibody (1:1000, Invitrogen). The hybridization signal was amplified with TSA Cy3 System (PerkinElmer). The *Nlgn1*, *Nlgn2*, and *Nlgn3* mRNA-positive granules colocalizing with FMRP-positive granules were visualized with confocal microscope LSM 700, Axio Imager Z2 (Zeiss) in a stack of 0.4 μm thick *Z* sections by frame scan mode using a 40×/1.30 Oil DIC M27 objective and the Zen software (Zeiss). In Fig. 1g–i and Fig. S1a–c, the single *Z* sections are presented with 1.2× zoom factor on frame size 1024 × 1024, so pixel size is 0.13 μm.

### Detection of Newly Synthesized Proteins in Synaptoneurosomes Using Click-iT Chemistry

To detect de novo protein synthesis, we used alkyne analog of puromycin, O-propargyl-puromycin (OP-puro, #NU-931-05, Jena Bioscience, Jena, Germany). OP-puro forms covalent conjugates with nascent polypeptide chains that can be captured by copper(I)-catalyzed azide-alkyne cycloaddition (Click-iT). Freshly isolated synaptoneurosomes from P30-40 WT mice were NMDAR-stimulated and incubated for 20 min at 37 °C. Digitonin (0.02%) was added to the synaptoneurosomes 3 min before the end of incubation. Afterwards, OP-puro was added to 50 μM concentration and the synaptoneurosomes were incubated at 37 °C for additional 3 min, to enable incorporation of OP-puro into nascent proteins. The controls were incubated without OP-puro. After stimulation and OP-puro labeling, synaptoneurosomes were resuspended in lysis buffer (50 mM Tris-HCl, pH 8.0, 1% SDS), and click reaction with TAMRA-azide or biotin-azide (Invitrogen) was performed accordingly to the manufacturer’s protocol. After reaction with TAMRA-azide, the proteins were precipitated with methanol and the pellets were dissolved in Laemmli buffer containing 100 mM DTT and boiled for 10 min at 98 °C. Samples were run on SDS-PAGE and visualized fluorescently using Amersham Imager 600 (GE Healthcare, Wauwatosa, WI).

In the case of click reaction with biotin-azide, the proteins were precipitated with methanol, and the pellets were dissolved in 4% SDS and sonicated using Bioruptor Plus (6× 15 s high/ 30 s off or until a clear solution was obtained). Next, the samples were diluted with PBS to 0.4% SDS final concentration. Input samples (5%) were collected, mixed with 2× Laemmli buffer with 100 mM DTT, boiled for 5 min, and retained for running SDS-PAGE. OP-puro-biotin-labeled proteins were enriched on streptavidin Dynabeads (MyOne; Invitrogen) preequilibrated in 0.4% SDS in PBS. Samples were incubated with the streptavidin beads for 1 h at RT and subsequently at 4 °C overnight. Then, the beads were washed in the following sequence: 3× 0.2% SDS in PBS, 2× urea buffer (8 M urea, 100 mM Tris pH 8.0, 200 mM NaCl), and 2× PBS. The proteins were eluted in Laemmli buffer containing 100 mM DTT and 2.5 mM biotin for 10 min at 98 °C and newly synthesized proteins were visualized by western blotting with anti-NLGN1, NLGN2, and NLGN3 antibodies (Synaptic Systems, as below).

### Polyribosomes Profiling, RNA Isolation, and qRT-PCR

The polyribosome fractionation was performed accordingly to the detailed protocol published recently [38]. Synaptoneurosomes prepared from hippocampi and a part of adjacent cerebral cortex of 2-month-old male (P60, WT, and *Fmr1* KO mice) were NMDAR-stimulated and incubated for 20 min. Next, synaptoneurosomes were lysed in the buffer A (20 mM Tris-HCl pH 7.4, 125 mM NaCl, 10 mM MgCl_2_, 2 mM DTT, 200 μg/ml cycloheximide, 120 U/ml RiboLock RNase Inhibitor, and protease inhibitor cocktail) containing 1.5% IGEPAL CA-630 (Sigma-Aldrich) and the membranous structures were removed by spinning at 20000 rcf for 15 min at 4 °C. The supernatant was loaded on 10–50% *w*/*w* linear sucrose gradient prepared in buffer A and spun at 38000 rpm for 2 h in TH641 rotor in Sorvall WX Ultra Series Centrifuge (Thermo Scientific). Each gradient was separated into 23 fractions (500 μl each) which were combined into three fractions: messenger ribonucleoprotein complexes and monosome (mono), light polysomes (L-poly), and heavy polysomes (H-poly) accordingly to polyribosomes absorbance profile monitored at 254 nm using ISCO UA-6 UV/VIS detector. Then, each fraction was supplemented with linear acrylamide (20 μg/ml) and 10 ng of in vitro transcribed fragment of *Arabidopsis thaliana LSm* gene as a spike control. Next, the total RNA was extracted at − 80 °C overnight with 1:10 volume of sodium acetate (3 M, pH 5.2), 40 μg glycogen as precipitation enhancer, and one volume of isopropanol. After centrifugation, the pellets were dissolved in proteinase K digestion buffer (0.5% SDS, 10 mM EDTA, 50 mM Tris-HCl. pH 7.4), and proteinase K was added to final concentration of 100 μg/ml for 20 min incubation at 42 °C with shaking. Next, RNA was extracted with two volumes of phenol/chloroform/isoamyl alcohol (25:24:1) solution (Sigma-Aldrich) in Phase lock Gel Heavy 2 ml tubes (5 Prime, San Fransisco, CA). The RNA from aqueous phase was extracted again with one volume of chloroform and precipitated overnight with 2.5 volume of 96% ethanol and 1:10 volume of 3 M sodium acetate, pH 5.2 in − 80 °C. After centrifugation, the pellets were washed twice in 70% ethanol, once in 90% ethanol, air-dried for about 7 min, and dissolved in 30 μl of RNase-free water. Concentration and quality of extracted RNA was checked using DS-11 Spectrophotometer (DeNovix). The RNA was reverse transcribed using random primers (GeneON, #S300, 200 ng per reaction) and SuperScript IV Reverse Transcriptase (Thermo Scientific). The qRT-PCR was performed using Light Cycler 480 Probes Master Mix (Roche) and TaqMan primer/probes for *Nlgn1*, *Nlgn2*, *Nlgn3*, *Gapdh* as above, and *LSm* (At02174020_g1, Thermo Scientific) in 15 μl final volume. Relative mRNA levels in different fractions were determined using the ∆Ct (where Ct is threshold cycle) relative quantification method and presented as the % of mRNA in each fraction. The values were normalized to *LSm* spike-in control. Statistical analysis from four independent experiments (*n* = 4; about 100 mg tissue per condition) was performed using one-way (for analysis within genotype) and two-way (for comparisons between genotypes) ANOVA followed by Sidak’s multiple comparisons test in GraphPad Prism version 7.03.

### qRT-PCR

For qRT-PCR (Fig. S2), total RNA was extracted from male P30-40 WT and *Fmr1* KO synaptoneurosomes with TRIzol Reagent (Ambion). 1 μg of RNA was reverse transcribed using random primers (GeneON, #S300, 200 ng per reaction) and SuperScript IV Reverse Transcriptase (Thermo Scientific). The qRT-PCR was performed using Light Cycler 480 Probes Master Mix (Roche) and TaqMan primer/probes for *Nlgn1*, *Nlgn2*, *Nlgn3*, and *Gapdh* as above. The values were calculated using *Gapdh* for normalization and 2(-ddCt) method for relative quantification; *n* = 6 mice/genotype.

### BS^3^ Cross-Linking of Surface Proteins in Synaptoneurosomes and Hippocampal Cultures

The basal state and NMDAR-stimulated synaptoneurosomes from male P40-50 WT and *Fmr1* KO mice were subjected to cross-linking (basal state, 2.5, 5, 10, and 20 min after the stimulation) using 2 mM BS^3^ (bis(sulfosuccinimidyl)suberate, #21580, Thermo Scientific), the membrane-impermeable cross-linker which links the cell surface-expressed proteins [44]. BS^3^ was dissolved in freshly prepared 5 mM sodium citrate, pH 5.0. The cross-linking reaction was carried for 30 min on ice and quenched by adding 100 mM glycine for 10 min. Then, the samples were snap frozen on dry ice and stored at − 80 °C before immunoblotting. The results of cross-linking in synaptoneurosomes were quantified from six independent experiments (*n* = 6; Figs. 4, 5, and 6).

For cross-linking of proteins in mouse hippocampal cultures (DIV19), the cells were washed twice with chelating buffer (1 mM EDTA in HBSS) to disrupt the Ca^2+^-dependent NLGNs-neurexins interactions [15, 45]. 2 mM BS^3^ in HBSS was applied for 30 min on ice and 100 mM glycine was spiked for 10 min afterwards [44]. Cells were lysed in 1% SDS, 50 mM Tris-HCl, and 150 mM NaCl with protease inhibitor cocktail (Roche), sonicated using Bioruptor Plus (2× 5 s high/ 30 s off), centrifuged at 20000 rcf for 10 min at 4 °C and protein concentration was measured in supernatant SDS extracts using Pierce BCA protein assay (Thermo Scientific) before western blotting. The results of cross-linking in hippocampal cultures were quantified from *n* = 12 (Figs. 4h and 5f).

### Surface Proteins Biotinylation

The freshly isolated synaptoneurosomes from male P40-50 WT and *Fmr1* KO mice were treated with 5 mM EZ-Link Sulfo-NHS-LC-Biotin (#21335, Thermo Scientific) dissolved in HB, pH 8.0 for 30 min at 4 °C. Then, the samples were centrifuged at 1000 rcf for 10 min at 4 °C and the pellet of synaptoneurosomes containing biotinylated surface-expressed proteins was washed twice before lysis in 1% TritonX-100 in PBS, pH 7.4. Then, the extracts were diluted 50× with lysis buffer and combined with 50 μl (0.5 mg) of washed Dynabeads MyOne Streptavidin T1 (Invitrogen). The binding of the beads to biotinylated surface proteins was performed for 2 h at 4 °C while rotating. Afterwards, the beads were washed three times with lysis buffer and denatured for 10 min at 98 °C with Laemmli buffer containing 100 mM DTT and 2.5 mM biotin (Sigma-Aldrich). The eluted samples were used for western blotting. Six independent experiments were performed (*n* = 6; Fig. 4j, 5h, and 6f).

### Deglycosylation

Deglycosylation of the cross-linked samples was performed according to the manufacturer’s protocols using the glycosidase EndoH (endoglycosidase H) and PNGase F (peptide N-glycosidase F; New England Biolabs, Ipswich, MA).

### SDS-PAGE and Western Blotting

For SDS-PAGE, the same amount of protein samples was separated on 7.5% (for cross-linked proteins) and 10 or 12% (to observe cleaved NLGNs C-terminal fragments—cNLGNs CTF) TGX Stain-Free FastCast Acrylamide gels (Bio-Rad). The gels were electro-transferred onto 0.45 μm PVDF membrane (Immobilon-P, Merck Millipore) using 25 V for 5 or 7 min in Trans-Blot Turbo Transfer System (Bio-Rad) in 25 mM Tris, 192 mM glycine, 20% ethanol, or, in the case of cross-linked proteins, 25 V for 1 h 30 min in Trans-Blot SD Semi-Dry Transfer Cell (Bio-Rad) in 300 mM Tris, 298 mM glycine, 3.5 mM SDS, 20% ethanol. The equal protein loading and transfer was controlled each time by imaging the gel with Gel Doc XR+ Gel Documentation System (Bio-Rad) before and after protein transfer. The membranes were blocked with 5% BSA and 3% normal goat serum (NGS) in PBST for 1 h at RT. The incubation with the following dilutions of primary antibodies was performed overnight at 4 °C with gentle shaking: 1:1000 mouse anti-NLGN1 extracellular domain (#129111, Synaptic Systems, Goettingen, Germany, RRID:AB_887747); 1:1000 rabbit anti-NLGN1 cytoplasmic domain (#129013, Synaptic Systems, RRID:AB_2151646); 1:1000 mouse anti-NLGN2 cytoplasmic domain (#129511, Synaptic Systems, RRID:AB_2619813); 1:2000 rabbit anti-NLGN3 cytoplasmic domain (#129113, Synaptic Systems, RRID:AB_2619816); 1:1000 mouse anti-β3-Tubulin (#4466, Cell Signaling, RRID:AB_1904176). Rabbit anti-FMRP 1:1000 (#7104S, Cell Signaling, RRID:AB_10950502) was used to test RNA co-immunoprecipitation efficiency. Antibodies used for excitatory synapses markers in male P30-40 WT homogenates and synaptoneurosomes were mouse anti-PSD-95 (1:500, MAB1598, Merck Millipore, RRID:AB_11212185), rabbit anti-VGLUT2 (1:5000, #135403, Synaptic Systems, RRID:AB_887883), rabbit anti-NLGN1 (1:1000, #129013, Synaptic Systems), and rabbit anti-NLGN3 (1:1000, #129113, Synaptic Systems), while markers for inhibitory synapses were mouse anti-GEPHYRIN (1:1000, #147111, Synaptic Systems, RRID:AB_887719), mouse anti-VGAT (1:1250, #131011, Synaptic Systems, RRID:AB_887872), and mouse anti-NLGN2 (1:1000, #129511, Synaptic Systems), and for additional loading control mouse anti-GAPDH (1:1000, #MAB374, Merck Millipore, RRID:AB_2107445) was used. After washing 3× in PBST, the membranes were incubated with peroxidase-labeled secondary antibodies (Vector Laboratories, Burlingame, CA) diluted 1:10000 in 5% BSA in PBST for 1 h at RT and washed again 3× in PBST. The protein bands were visualized by enhanced chemiluminescence using Amersham ECL Prime Western Blotting Detection Reagent (GE Healthcare) and Amersham Imager 600 (GE Healthcare). Some of the membranes were incubated in the stripping buffer (0.1 M glycine, 2% SDS, pH 3.0) for 20 min at room temperature, washed 3× with PBST before blocking as described above and reprobed with another primary antibody produced in different host than already used one. The intensities of pixels corresponding to the specific protein levels were quantified using the ImageJ software (RRID:SCR_003070) and the background of each western blot was subtracted.

In experiments where protein levels were compared between the genotypes, *Fmr1* KO measures were relativized to WT basal state (Bsl) values (Fig. 3a–c *n* = 6; Fig. 3d–f *n* = 5–9). Statistical analysis was performed using two-tailed unpaired Student’s *t* test for comparisons between genotypes and paired Student’s *t* test for comparisons within the genotype in GraphPad Prism version 7.03 (GraphPad Software Inc.; RRID:SCR_002798).

## Results

### FMRP Interacts with *Nlgn1*, *Nlgn2*, and *Nlgn3* mRNAs

To date, two independent studies reported FMRP interaction with *Nlgns* mRNAs [29, 46]. The results of high-throughput sequencing of RNAs isolated by cross-linking immunoprecipitation (HITS-CLIP) have shown that FMRP can interact with *Nlgn2* and *Nlgn3* mRNAs in mouse brain [29], while others have shown the association of *Nlgn1* and *Nlgn2* mRNAs with FMRP in whole brain extracts [46]. To corroborate these results, we employed the model that allows for studying mRNA association with FMRP at the synapse. We used synaptoneurosomes, biochemical preparations enriched in pre- and postsynaptic proteins commonly used to study local translation of synaptic mRNAs and described by us and others before [38–41, 43, 47]. Synaptoneurosomal protein extracts isolated from WT and *Fmr1* KO mice were used for RNA co-immunoprecipitation with anti-FMRP 7G1-1 antibody. As shown in Fig. 1a, FMRP was precipitated from WT synaptoneurosomes, while it was not detected in *Fmr1* KO immunoprecipitates. After RNA extraction from immunoprecipitated samples, we performed qRT-PCR to assess the levels of FMRP-associated *Nlgn1*, *Nlgn2*, *Nlgn3*, and control mRNAs. *Psd-95* mRNA, a known FMRP target [36, 48], was used as a positive control (3.51-fold increase, *p* < 0.0001, *t* = 8.413, df = 12; Fig. 1b), while *Gapdh* mRNA served as a negative control of the experiment (Fig. 1c). As shown in Fig. 1d–f, *Nlgn1*, *Nlgn2*, and *Nlgn3* mRNAs were significantly enriched in immunoprecipitates from WT mice when compared to *Fmr1* KO mice (*Nlgn1*: 2.77-fold increase, *p* = 0.0239, *t* = 2.583, df = 12; *Nlgn2*: 3.2-fold increase, *p* = 0.0051, *t* = 3.415, df = 12; and *Nlgn3*: 2-fold increase, *p* = 0.0067, *t* = 3.629, df = 8 in WT FMRP IP versus *Fmr1* KO FMRP IP by one-way ANOVA followed by post hoc Sidak’s multiple comparisons test, *n* = 3–4).

To additionally confirm the interaction of *Nlgn1*, *Nlgn2*, and *Nlgn3* mRNAs with FMRP in dendrites, we studied their colocalization in cultured hippocampal neurons (DIV21). Endogenous *Nlgn1*, *Nlgn2*, and *Nlgn3* mRNAs were revealed by fluorescence in situ hybridization combined with immunodetection to visualize FMRP protein (Fig. 1g–i). *Nlgn1*, *Nlgn2*, and *Nlgn3* mRNAs were localized in granules along the proximal dendrites and exhibited partial colocalization with FMRP (visualized as double fluorescent yellow dots; Fig. 1g–i). FISH sense riboprobes were used as a negative control of the experiment (Fig. S1a–c). Taken together, our results from RNA co-IP and FISH-IF experiments indicate that FMRP can associate with mRNAs of studied *Nlgn1*, *Nlgn2*, and *Nlgn3* in dendrites and at the synapse.

### Activity-Induced Translation of *Nlgn1*, *Nlgn2*, and *Nlgn3* mRNAs at the Synapse Is Regulated by FMRP

**Fig. 2:**
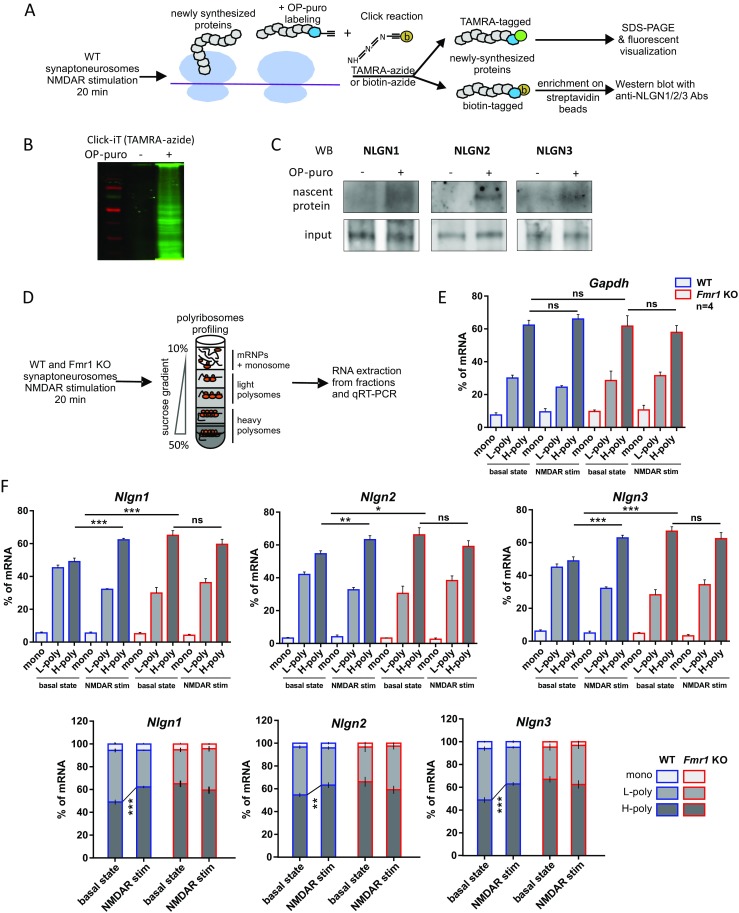
Activity-induced translation of *Nlgn1*, *Nlgn2*, and *Nlgn3* mRNAs occurs locally at the synapse and is regulated by FMRP. **a**–**c** Detection of de novo protein synthesis in synaptoneurosomes using alkyne analog of puromycin (OP-puro) tagging and Click chemistry. **a** Scheme of the experiments. **b** OP-puro-labeled proteins detected by click reaction with TAMRA-azide and fluorescently visualized in polyacrylamide gel. **c** Western blot detection of nascent NLGN1, NLGN2, and NLGN3 proteins in synaptoneurosomes isolated from WT mice. OP-puro-biotin-tagged proteins were enriched on streptavidin beads. Input fraction served as the experiment’s control. **d** Workflow of synaptoneurosomes’ polyribosomes profiling experiments. The untreated (basal state) or NMDAR-stimulated synaptoneurosomes from WT and *Fmr1* KO mice were lysed, separated in sucrose density gradient, and, according to the absorbance profile, divided into three fractions: free messenger ribonucleoprotein complexes and monosome (mono), light polysomes (L-poly), and heavy polysomes (H-poly), the latter corresponding to the actively translating polyribosomal fraction. **e**–**f** Polyribosomes association of **e** *Gapdh*, **f** *Nlgn1*, *Nlgn2*, and *Nlgn3* mRNAs isolated from unstimulated and NMDAR-stimulated WT and *Fmr1* KO synaptoneurosomes assessed by qRT-PCR. The abundance of *Nlgn1*, *Nlgn2*, *Nlgn3*, and *Gapdh* mRNAs in each of the analyzed fractions is presented as the separated percentage (top panel) and the stacking percentage (bottom panel) of total *Nlgn* mRNAs present in all three fractions. Graphs represent mean values ± SEM, *n* = 4, *p < 0.05, **p < 0.01, ***p < 0.0001 by one-way ANOVA for comparisons within a genotype and two-way ANOVA for comparisons between genotypes, both followed by post hoc Sidak’s multiple comparisons test; ns, not significant

Based on the immunoprecipitation data indicating the direct binding of FMRP to *Nlgn1*, *Nlgn2*, and *Nlgn3* mRNAs, we hypothesized that FMRP can regulate their synaptic translation. To detect the nascent neuroligin polypeptides synthesized in synaptoneurosomes in response to stimulation, we used the analogue of puromycin (OP-puro, O-propargyl-puromycin; Fig. 2a) that forms covalent conjugates with newly synthesized polypeptide chains and can be next captured by click reaction. OP-puro-tagged proteins were detected by click reaction with TAMRA-azide and visualized in polyacrylamide gel (Fig. 2b). Next, the OP-puro-labeled proteins were clicked with biotin-azide and enriched on streptavidin beads. Western blots with anti-NLGN1, NLGN2, and NLGN3 antibodies were performed to visualize de novo synthesized proteins. As shown in Fig. 2c, we detected local synthesis of NLGN1, NLGN2, or NLGN3 in stimulated synaptoneurosomes. Western blots of input fractions (bottom panel) served as a control for the presence of NLGN1, NLGN2, or NLGN3 proteins in the studied synaptoneurosomal fractions.

To stimulate NMDARs on synaptoneurosomes, we decided to use the protocol that was previously shown to induce local translation of proteins [36, 42, 49]. For example Mudashetty et al. (2007) reported that rapid NMDAR-induced translation of FMRP-dependent mRNAs observed in wild-type mouse synaptoneurosomes is absent in *Fmr1* KO mice, what suggests translational impairments in *Fmr1* KO.

To show that local translation of *Nlgns* mRNAs is FMRP-dependent, we investigated its potential dysregulation in the mouse model of FXS, *Fmr1* KO mice. To this end, we studied the association of synaptic *Nlgn1*, *Nlgn2*, and *Nlgn3* mRNAs with translating polyribosomes by polyribosome profiling [38]. WT and *Fmr1* KO synaptoneurosomes were either untreated or NMDAR-stimulated for 20 min [38, 42]. Next, synaptoneurosomes were lysed and fractionated on linear sucrose gradient. The collected polysomal fractions were divided into three groups, which represent the free messenger ribonucleoprotein complexes and monosome (mono), light polysomes (L-poly), and heavy polysomes (H-poly), the latter corresponding to the actively translating polyribosomal fraction (Fig. 2d). Each fraction was supplemented with the same amount of spike RNA as a control for normalization. After total RNA extraction from all fractions, qRT-PCR was performed to assess the activity-dependent distribution of *Nlgns* mRNAs on polyribosomes. The stimulation of WT synaptoneurosomes resulted in a significant shift of *Nlgn1*, *Nlgn2*, and *Nlgn3* mRNAs towards heavier polysomal fractions, characteristic for active translation (shift in mRNA percentage in H-poly fraction of WT basal state versus WT NMDAR-stimulated synaptoneurosomes: 49 to 62% of *Nlgn1* mRNA, *p* < 0.0001, *t* = 7.74, df = 18; 55 to 63% of *Nlgn2* mRNA, *p* = 0.0012, *t* = 3.852, df = 18; 49 to 63% of *Nlgn3* mRNA, *p* < 0.0001, *t* = 6.158, df = 18 by one-way ANOVA followed by post hoc Sidak’s multiple comparisons test, *n* = 4; Fig. 2f). The association of the control *Gapdh* mRNA with polyribosomes was not changed upon the stimulation and between two analyzed genotypes (Fig. 2e). The polysome profile was significantly altered in *Fmr1* KO synaptoneurosomes as compared to WT showing increased association of *Nlgn1*, *Nlgn2*, and *Nlgn3* mRNAs with actively translating heavy polyribosomes in *Fmr1* KO. Moreover, in *Fmr1* KO synaptoneurosomes the stimulation of NMDARs did not increase the translation rates of *Nlgn1*, *Nlgn2*, and *Nlgn3* mRNAs (Fig. 2f). In conclusion, the absence of FMRP leads to translational disinhibition of *Nlgns* mRNAs what results in their enhanced translation that cannot be further increased by the stimulation. The levels of *Nlgns* mRNAs were the same in both WT and *Fmr1* KO synaptoneurosomes (Fig. S2), thus excluding transcriptional effects.

### Elevated Levels of NLGN1 and NLGN3 in *Fmr1* KO Synaptoneurosomes and Hippocampal Cultures

**Fig. 3:**
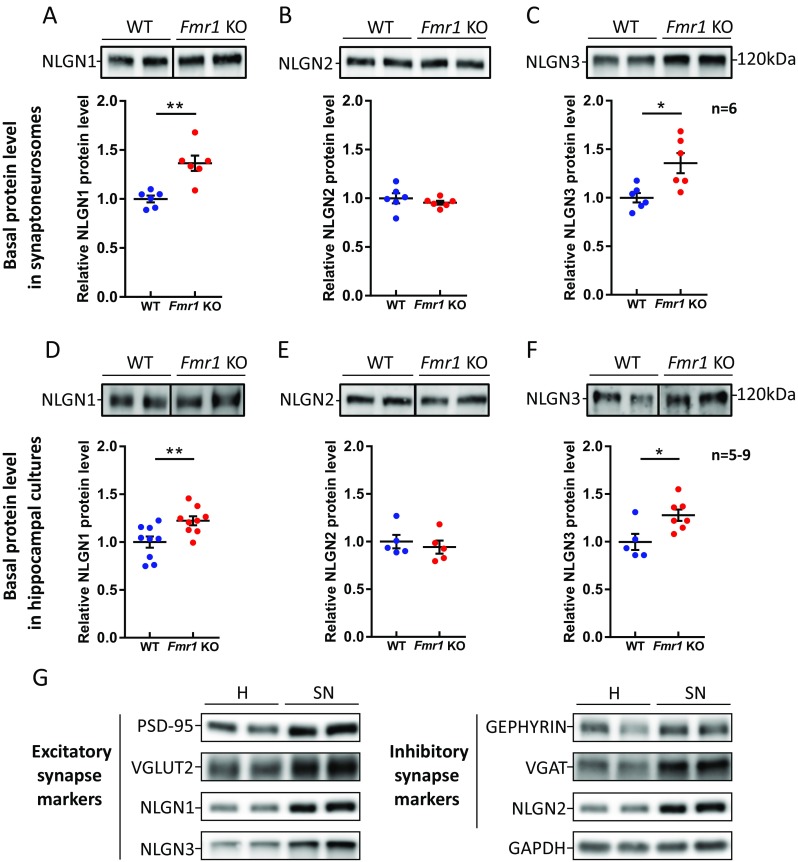
The level of NLGN1 and NLGN3 proteins is elevated in synaptoneurosomes and hippocampal cultures from *Fmr1* KO mice. **a**–**c** Western blot analysis of **a** NLGN1, **b** NLGN2, and **c** NLGN3 protein levels in synaptoneurosomes isolated from WT and *Fmr1* KO mice. Below, the graphs representing mean values ± SEM from densitometric analysis of immunoblots, *n* = 6 mice/genotype, NLGN1 *p* = 0.0016; NLGN2 *p* = 0.4293 (not significant); NLGN3 *p* = 0.0112; *p < 0.05, **p < 0.01 by unpaired Student’s *t* test. **d**–**f** Western blot analysis of **d** NLGN1, **e** NLGN2, and **f** NLGN3 protein levels in DIV21 WT and *Fmr1* KO hippocampal cultures. Below, the graphs representing mean values ± SEM from densitometric analysis of immunoblots, *n* = 5–9, NLGN1 *p* = 0.0086; NLGN2 *p* = 0.577 (not significant); NLGN3 *p* = 0.0189; *p < 0.05, **p < 0.01, by unpaired Student’s *t* test. For protein loading control, see Fig. S3. **g** Comparative western blots presenting excitatory and inhibitory synapses’ markers in brain homogenates (H) and synaptoneurosomes (SN) from WT mice

As a result of altered *Nlgns* mRNA translation at the *Fmr1* KO synapses, we expected to detect more NLGN proteins in isolated synaptoneurosomes. To test this hypothesis, we performed western blots on synaptoneurosomal protein extracts and we observed an increase in NLGN1 and NLGN3 protein level in *Fmr1* KO (36% increase of NLGN1, *p* = 0.0016, *t* = 4.3, df = 10 and 36% increase of NLGN3, *p* = 0.0112, *t* = 3.101, df = 10 by unpaired Student’s *t* test, *n* = 6; Fig. 3a,c and protein loading in Fig. S2a,c–e). However, no difference in protein level was detected for NLGN2 when WT and *Fmr1* KO synaptoneurosomes were compared (*p* = 0.4293, *t* = 0.824, df = 10 by unpaired Student’s *t* test, *n* = 6; Fig. 3b and protein loading in Fig. S2b). The level of mRNAs for *Nlgn1*, *Nlgn2*, and *Nlgn3* was validated in synaptoneurosomes using qRT-PCR and no differences were detected between WT and *Fmr1* KO indicating for dysregulated local protein synthesis (Fig. S2).

These results were further corroborated in cultured hippocampal neurons (DIV21). The level of NLGN1 and NLGN3 was augmented in neuronal cell cultures prepared from *Fmr1* KO mice when compared with WT (22% increase of NLGN1, *p* = 0.0086, *t* = 2.992, df = 16 and 28% increase of NLGN3, *p* = 0.0189, *t* = 2.796, df = 10 by unpaired Student’s *t* test, *n* = 5–9; Fig. 3d, f and protein loading in Fig. S2f, h–j). Here again, we did not detect the changes in NLGN2 level between the two analyzed genotypes (*p* = 0.5774, *t* = 0.5807, df = 8 by unpaired Student’s *t* test, *n* = 5; Fig. 3e and protein loading in Fig. S2g). Since the studied NLGN1, NLGN2, and NLGN3 are located on both excitatory and inhibitory synapses, we checked whether our synaptoneurosomal preparations contain both types of synaptic markers. Using the antibodies recognizing excitatory (PSD-95, VGLUT2, NLGN1, NLGN3) and inhibitory synapses (gephyrin, VGAT, NLGN2), we have shown that they are equally enriched in synaptoneurosomes when compared with brain homogenates. That would mean that since in the brain there is less of inhibitory synapses, there is equally less of them in synaptoneurosomes (Fig. 3g).

### Enhanced Postsynaptic Membrane Targeting of NLGN1 and NLGN3 at *Fmr1* KO Synapses

Elevated levels of NLGN1 and NLGN3 in *Fmr1* KO synaptoneurosomes raised the question about their intracellular distribution and abundance on the surface of the synapse. NLGNs function as homo- and heterodimers and their dimerization occurs prior to cell surface incorporation [15]. To distinguish NLGNs dimers (both homo- and heterodimers) residing on the synaptic membrane from the cytoplasmic monomers, we used chemical in situ cross-linking with BS^3^ reagent which does not penetrate the cell membrane and therefore cross-links only the surface proteins. This approach was developed by Poulopoulos et al. [15] to study NLGNs’ homo- and heterodimer complexes in cultured primary neurons and adapted by us to distinguish the surface NLGN1, NLGN2, and NLGN3-containing homo- and heterodimers from intracellular monomers in WT and *Fmr1* KO synaptoneurosomes. Using this method, we could observe the dynamics of NLGNs shuttling induced by NMDAR activation [22, 50, 51]. In all experiments, the synaptoneurosomes were either unstimulated (to assess the basal NLGNs levels) or NMDAR-stimulated (2.5, 5, 10, and 20 min) and cross-linked.

#### Neuroligin 1

**Fig. 4:**
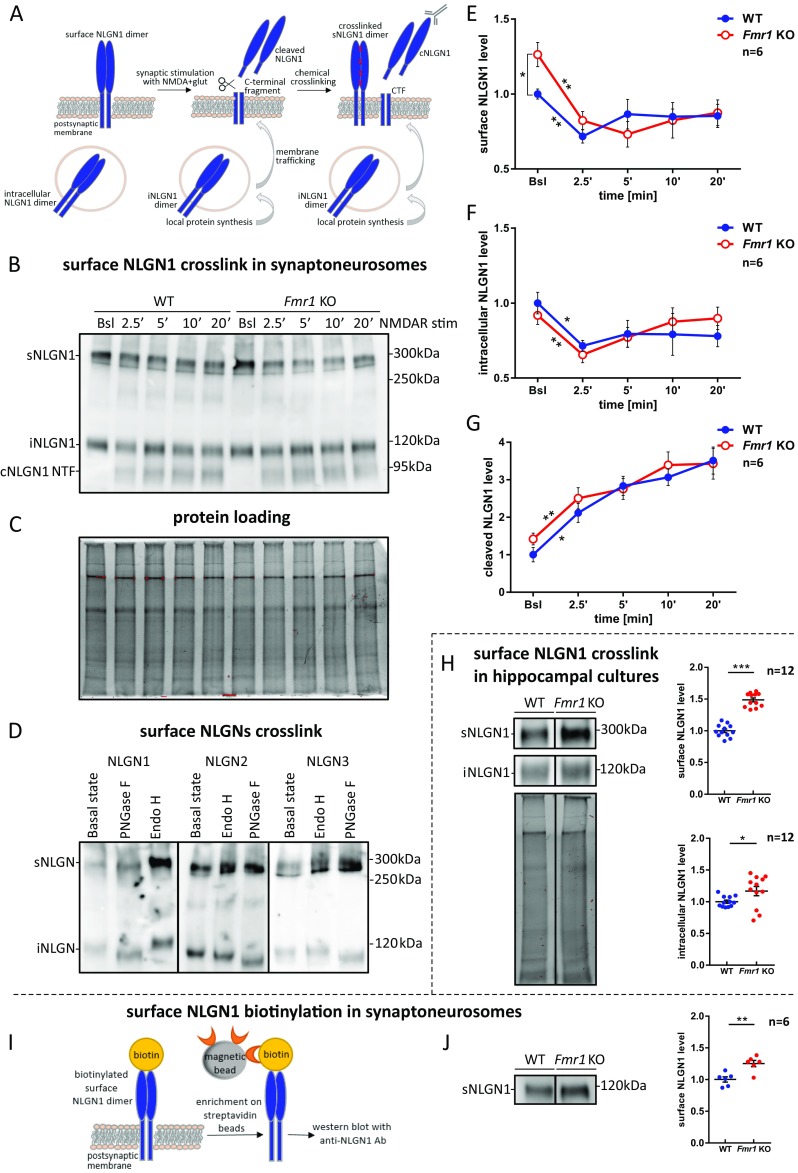
Enhanced postsynaptic membrane targeting of NLGN1 and its activity-dependent cleavage in synaptoneurosomes from WT and *Fmr1* KO mice. **a** Schematic representation of NLGN1 local protein synthesis, membrane trafficking and proteolytic cleavage in response to the stimulation. NLGN1 species detected on the western blot after cross-linking experiment are depicted. **b** Western blot with anti-NLGN1 antibody recognizing NLGN1 extracellular domain. Cross-linked NLGN1 surface dimers (sNLGN1) are detected at about 300 kDa (corresponding to homo- or heterodimers containing NLGN1), intracellular NLGN1 monomers (iNLGN1) at about 120 kDa and cleaved N-terminal fragment of NLGN1 (cleaved NLGN1 NTF) at about 95 kDa. **c** TGX acrylamide gel visualization by Bio-Rad Gel Doc XR+ to confirm equal protein loading on the gel. **d** Western blot showing deglycosylation of cross-linked NLGN1, NLGN2, and NLGN3 proteins with PNGase F and Endo H. **e***–***g** Densitometric quantification of the western blot bands from independent experiments, *n* = 6. The graphs represent the mean values ± SEM of **e** surface, **f** intracellular, and **g** cleaved NLGN1 protein levels relative to WT untreated sample (WT Bsl), *p < 0.05, **p < 0.01 by unpaired Student’s *t* test for comparisons between genotypes and paired Student’s *t* test for comparisons within the genotype. **h** Western blot showing surface (sNLGN1) and intracellular (iNLGN1) NLGN1 protein levels in cross-linked DIV19 hippocampal cultures. Below, the protein loading. The graphs represent mean values ± SEM from densitometric analysis of immunoblots, *n* = 12; *p < 0.05, ***p < 0.001 by unpaired Student’s *t* test. **i** Scheme of biotinylation experiment. **j** Immunoblot probed with anti-NLGN1 antibody showing biotinylated surface NLGN1 (sNLGN1) level in WT and *Fmr1* KO synaptoneurosomes. The graph shows densitometric quantification of the bands from six independent experiments ± SEM, *n* = 6; **p < 0.01, by unpaired Student’s *t* test

On the western blots, we were able to observe cross-linked surface NLGN1-containing dimers (sNLGN1 at about 300 kDa) and intracellular NLGN1 monomers (iNLGN1 at about 120 kDa; Fig. 4a–c). The cross-linked NLGN1-containing dimers appeared at higher molecular weight than expected, probably due to the glycosylation of NLGN1. The enzymatic digestion of synaptoneurosomal protein extracts with PNGase F lowered NLGNs monomer bands on the western blot (Fig. 4d).

The densitometric analysis of western blots revealed higher basal level of cross-linked surface NLGN1-containing dimers in *Fmr1* KO synaptoneurosomes when compared to WT (26% increase, *p* = 0.0136, *t* = 2.988, df = 10 by unpaired Student’s *t* test, *n* = 6; Fig. 4e). The level of intracellular species of NLGN1 did not differ between genotypes in basal state (Fig. 4f). In cultured hippocampal neurons we observed higher abundance of cross-linked surface and intracellular NLGN1 in *Fmr1* KO cells when compared to WT (49% increase for sNLGN1, *p* < 0.0001, *t* = 11.45, df = 22 and 16% increase for iNLGN1, *p* = 0.0398, *t* = 2.185, df = 22 by unpaired Student’s *t* test, *n* = 12; Fig. 4h).

Interestingly, upon NMDAR stimulation of both WT and *Fmr1* KO synaptoneurosomes, we detected the appearance of additional bands at about 95 kDa on western blots (Fig. 4b). Since the antibody used for immunodetection in this experiment recognizes the extracellular domain of NLGN1, we assumed that these bands correspond to the cleaved N-terminal fragments of NLGN1 (cNLGN1 NTF at about 95 kDa), as reported by Peixoto et al. and Suzuki et al. [52, 53]. The induced proteolysis was accompanied by a significant decrease in the intensity of cross-linked surface NLGN1-containing bands as early as 2.5 min after NMDAR stimulation of both WT and *Fmr1* KO synaptoneurosomes (*p < 0.05, **p < 0.01 by paired Student’s *t* test; Fig. 4e, g). This would suggest the activity-induced shedding of surface NLGN1-containing dimers. Concurrently, the intracellular pool of NLGN1 was also diminished upon NMDAR stimulation for 2.5 min in both genotypes, probably due to its enhanced surface trafficking and incorporation to the postsynaptic density (*p < 0.05, **p < 0.01 by paired Student’s *t* test; Fig. 4f). Importantly, we have not observed differences in the intensity of proteolytic NLGN1 cleavage between WT and *Fmr1* KO mice (Fig. 4g). We assume that the changes in proportions observed for surface and intracellular NLGN1-containing species represent a dynamic process of NLGN1 activity-induced synaptic membrane incorporation and cleavage.

To further confirm the enhanced synaptic incorporation of NLGN1 into the postsynaptic membrane of *Fmr1* KO mice, we performed biotinylation of surface proteins, followed by streptavidin enrichment and western blot analysis (Fig. 4i). In this experiment, we confirmed the higher level of surface NLGN1 at *Fmr1* KO synapses in basal state when compared to WT (25% increase, *p* = 0.0331, *t* = 3.88, df = 10 by unpaired Student’s *t* test, *n* = 6; Fig. 4j).

#### Neuroligin 3

**Fig. 5:**
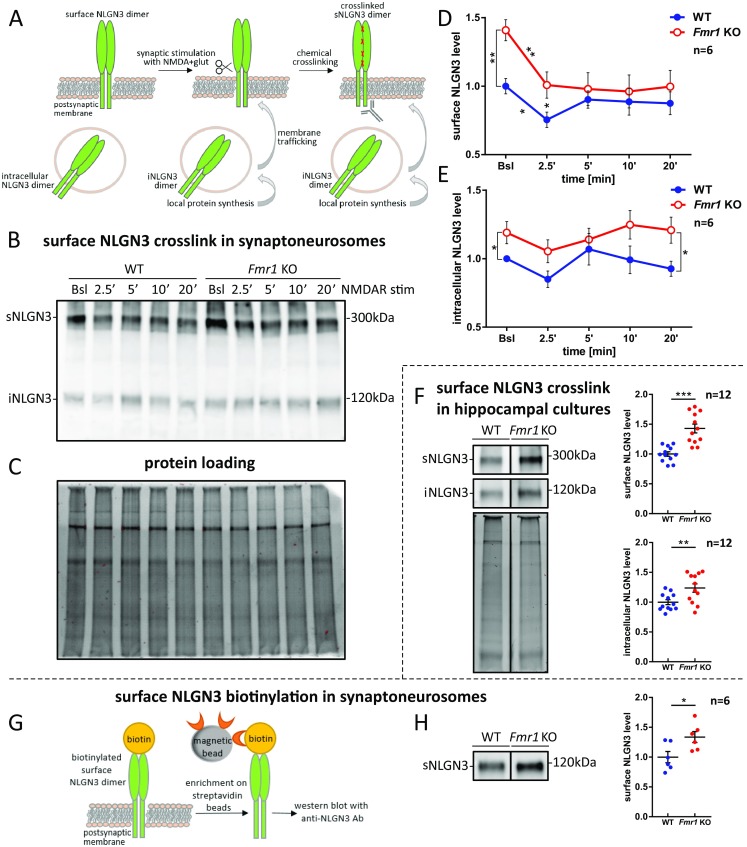
Enhanced postsynaptic membrane targeting of NLGN3 in *Fmr1* KO mice and reduction of its level upon the stimulation. **a** Schematic representation of NLGN3 local protein synthesis and membrane trafficking in response to the stimulation. NLGN3 species detected on the western blot after cross-linking experiment are depicted. **b** Western blot with anti-NLGN3 antibody recognizing NLGN3 cytoplasmic domain. Cross-linked NLGN3 surface dimers (sNLGN3) are detected at about 300 kDa (corresponding to homo- or heterodimers containing NLGN3), and intracellular NLGN3 monomers (iNLGN3) at about 120 kDa. **c** TGX acrylamide gel visualization by Bio-Rad Gel Doc XR+ to confirm equal protein loading on the gel used for immunoblots presented in **b** and Fig. 6b. The membrane was stripped and immunoprobed for NLGN2. **d**–**e** Densitometric quantification of the western blot bands from independent experiments, *n* = 6. Graphs represent the mean values ± SEM of **d** surface and **e** intracellular NLGN3 protein levels relative to WT unstimulated sample (WT Bsl), *p < 0.05, **p < 0.01 by unpaired Student’s *t* test for comparisons between genotypes and paired Student’s *t* test for comparisons within the genotype. **f** Western blot showing surface (sNLGN3) and intracellular (iNLGN3) NLGN3 protein levels in cross-linked DIV19 hippocampal cultures. Below, the protein loading. The graphs represent mean values ± SEM from densitometric analysis of immunoblots, *n* = 12; **p < 0.01, ***p < 0.001 by unpaired Student’s *t* test. **g** Scheme of biotinylation experiment. **h** Immunoblot probed with anti-NLGN3 antibody showing biotinylated surface NLGN3 (sNLGN3) level in WT and *Fmr1* KO synaptoneurosomes. The graph shows densitometric quantification of the bands from six independent experiments ± SEM, *n* = 6; *p < 0.05, by unpaired Student’s *t* test

The activity-controlled changes in abundance and distribution of synaptic NLGN3 were investigated using the same methods as for NLGN1 (Fig. 5). We performed the time course NMDARs stimulation and cross-linking of synaptoneurosomes, followed by western blots with antibody recognizing cytoplasmic domain of NLGN3 (Fig. 5a–c). We detected the significantly higher basal level of cross-linked NLGN3-containing dimers at the surface (sNLGN3 at about 300 kDa) of *Fmr1* KO synapses (41% increase, *p* = 0.0016, *t* = 24.274, df = 10 by unpaired Student’s *t* test, *n* = 6; Fig. 5d). Similarly to NLGN1, there was an activity-induced decrease in the level of surface NLGN3-containing dimers in both WT and *Fmr1* KO synaptoneurosomes (*p < 0.05, **p < 0.01 by paired Student’s *t* test; Fig. 5d), suggesting the proteolytic cleavage of NLGN3. Intracellular NLGN3 (iNGLN3 at about 120 kDa) level was increased in basal state *Fmr1* KO synaptoneurosomes (19% increase, *p* = 0.00457, *t* = 2.2281, df = 10 by unpaired Student’s *t* test, *n* = 6; Fig. 5e). We noticed a tendency for intracellular NLGN3 level to drop at 2.5 min after NMDAR stimulation of WT and *Fmr1* KO synaptoneurosomes (Fig. 5e). We also observed significantly faster recovery of intracellular NLGN3 pool 20 min after the stimulation of *Fmr1* KO synaptoneurosomes, probably because of generally higher rate of their synaptic translation (*p < 0.05 by unpaired Student’s *t* test, *n* = 6; Fig. 5e).

This results were further verified in cultured hippocampal neurons, where we also observed higher abundance of cross-linked surface and intracellular NLGN3 species in *Fmr1* KO cultures when compared to WT cultures (43% increase for sNLGN3, *p* < 0.0001, *t* = 5.154, df = 22 and 24% increase for iNLGN3, *p* = 0.0085, *t* = 2.888, df = 22 by unpaired Student’s *t* test, *n* = 12; Fig. 5f).

The analysis of biotinylated surface proteins confirmed that the abundance of synaptic membrane-tethered NLGN3 is significantly higher at *Fmr1* KO when compared to WT synapses in basal state (34% increase, *p* = 0.0287, *t* = 2.55, df = 10 by unpaired Student’s *t* test, *n* = 6; Fig. 5g, h).

#### Neuroligin 2

**Fig. 6:**
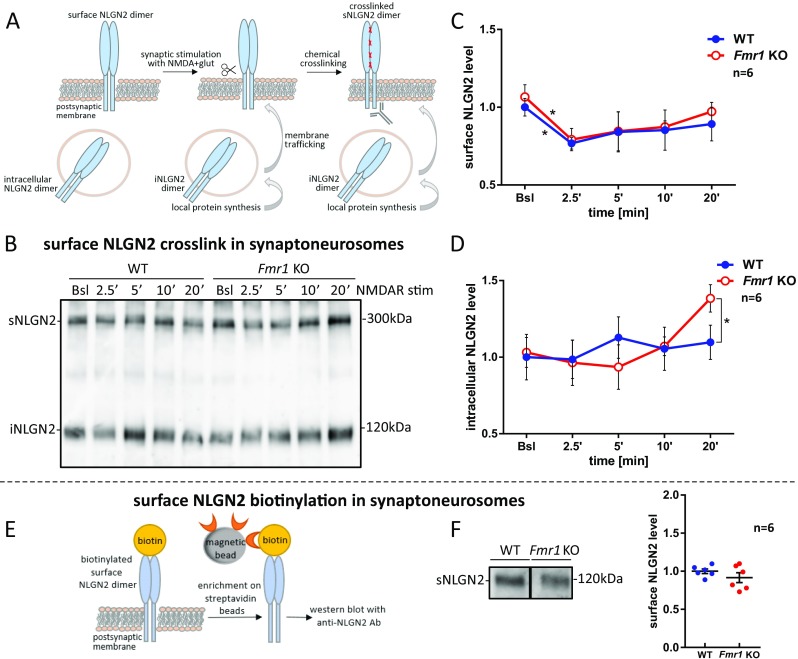
Postsynaptic membrane targeting of NLGN2 in WT and *Fmr1* KO mice and reduction of its surface level upon the stimulation. **a** Schematic representation of NLGN2 local protein synthesis and membrane trafficking in response to the stimulation. NLGN2 species detected on the western blot after cross-linking experiment are depicted. **b** Western blot with anti-NLGN2 antibody recognizing NLGN2 cytoplasmic domain. Cross-linked NLGN2 surface dimers (sNLGN2) are detected at about 300 kDa (corresponding to homo- or heterodimers containing NLGN2), and intracellular NLGN2 monomers (iNLGN2) at about 120 kDa. **c**–**d** Densitometric quantification of the western blot bands from independent experiments, *n* = 6. The graphs represent the mean values ± SEM of **c** surface and **d** intracellular NLGN2 protein levels relative to WT unstimulated sample (WT Bsl), *p < 0.05 by unpaired Student’s *t* test for comparisons between genotypes and paired Student’s *t* test for comparisons within the genotype. **e** Scheme of biotinylation experiment. **f** Immunoblot probed with anti-NLGN2 antibody showing biotinylated surface NLGN2 (sNLGN2) level in WT and *Fmr1* KO synaptoneurosomes. The graph shows densitometric quantification of the bands from six independent experiments ± SEM, *n* = 6

Next, we studied the activity-dependent distribution of cross-linked surface NLGN2-containing dimers (sNLGN2 at about 300 kDa) and intracellular NLGN2 (iNLGN2 at about 120 kDa), using the same experimental model and methods as for NLGN1 and NLGN3 (Fig. 6a). The in situ cross-linking assay, followed by western blot with anti-NLGN2 antibody recognizing its cytoplasmic domain did not reveal the difference in basal level of surface and intracellular NLGN2 between WT and *Fmr1* KO synaptoneurosomes (Fig. 6b–d). However, at 2.5 min after NMDAR stimulation, we observed a significant decrease in the level of surface NLGN2-containing dimers in both WT and *Fmr1* KO synaptoneurosomes (*p < 0.05 by paired Student’s *t* test, *n* = 6; Fig. 6c). These results suggest a possible proteolytic cleavage of NLGN2 upon NMDAR stimulation. The level of intracellular NLGN2 did not change significantly in response to the stimulation of WT synaptoneurosomes, although we observed the increase of iNLGN2 in *Fmr1* KO when compared to WT at 20 min after NMDAR stimulation (*p < 0.05 by unpaired Student’s *t* test, *n* = 6; Fig. 6d).

The biotinylation assay showed lack of differences in the surface NLGN2 level between WT and *Fmr1* KO mice in basal state (*p* = 0.2593, *t* = 1.2, df = 10 by unpaired Student’s *t* test, *n* = 6; Fig. 6e, f).

### Activity-Dependent Cleavage of NLGN1, NLGN2, and NLGN3 in Synaptoneurosomes

**Fig. 7:**
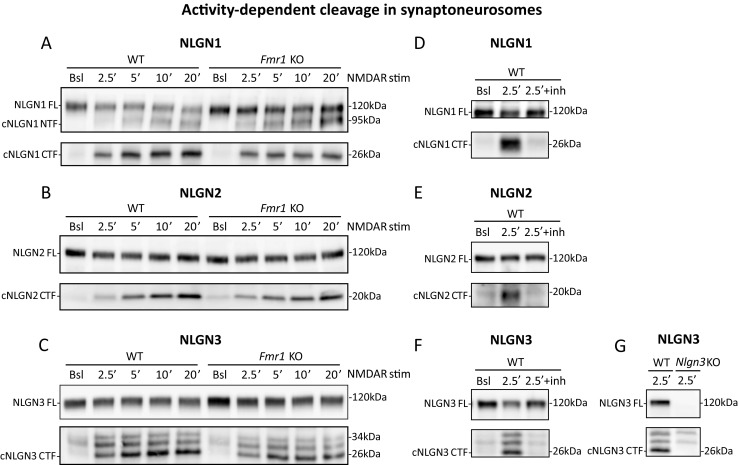
Activity-induced cleavage of NLGN1, NLGN2, and NLGN3 in WT and *Fmr1* KO synaptoneurosomes. **a**–**c** Representative western blots of **a** NLGN1, **b** NLGN2, and **c** NLGN3 in WT and *Fmr1* KO synaptoneurosomes either unstimulated (basal state, Bsl) or NMDAR-stimulated for 2.5, 5, 10, and 20 min. The bands at about 120 kDa represent the level of full-length NLGN1, NLGN2, and NLGN3. Cleaved N-terminal fragments of NLGN1 are detected at about 95 kDa and cleaved C-terminal fragments (cNLGNs CTF) at about 26 kDa for NLGN1, about 20 kDa for NLGN2, and about 26 kDa for NLGN3. **d**–**f** Incubation with MMP-9/-13 inhibitor I prevents NLGN1, NLGN2, and NLGN3 shedding as indicated by disappearance of cleaved NLGNs C-terminal fragments. Representative western blots showing full-length (NLGNs FL) and cleaved C-terminal fragments (cNLGNs CTF) of **d** NLGN1, **e** NLGN2, and **f** NLGN3 in WT synaptoneurosomes in basal state (Bsl) or 2.5 min after NMDAR stimulation (2.5′). In the case of MMP-9/-13 inhibitor I, samples were preincubated with the inhibitor (5 μm final conc.) 10 min before NMDAR stimulation (2.5′ + inh). **g** Verification of specific bands identified with anti-NLGN3 antibody using WT and *Nlgn3* KO synaptoneurosomes. Cleaved NLGN3 C-terminal fragment (cNLGN3 CTF) appears as a band with molecular weight of about 26 kDa

Our findings indicate that NMDAR stimulation provokes NLGNs dynamic shuttling at the synapse. In the cross-linking experiments, we observed proteolytic cleavage of NLGN1—the appearance of 95 kDa bands, followed by a simultaneous decrease in the amount of membrane-bound NLGN1-containing dimers, observed also for NLGN3 and NLGN2. This result suggested that all neuroligins can undergo activity-induced shedding. Therefore, we aimed to detect the products of their proteolytic cleavage with the use of antibodies recognizing C-terminal epitopes of NLGN1, NLGN2, and NLGN3. We observed the appearance of small (at about 20–26 kDa) C-terminal fragments of NLGN1, NLGN2, and NLGN3 (cNLGNs CTF) at 2.5, 5, 10, and 20 min after NMDAR stimulation of WT and *Fmr1* KO synaptoneurosomes (Fig. 7a–c and Fig. S4a–c). To prove that the observed bands are generated as a result of proteolysis, we incubated synaptoneurosomes with the inhibitor of matrix metalloproteinases (inhibitor I, #444252, Calbiochem) prior to NMDAR stimulation, and we observed the inhibition of neuroligins cleavage (Fig. 7d-f and Fig. S4d-f). *Nlgn3* knockout (*Nlgn3* KO) mice were used as a control for anti-NLGN3 antibody specificity. These preliminary results strengthen the data obtained in the cross-linking experiments. However, further experiments will be necessary to confirm the exact sites of proteolytic cleavage and responsible proteases.

## Discussion

**Fig. 8:**
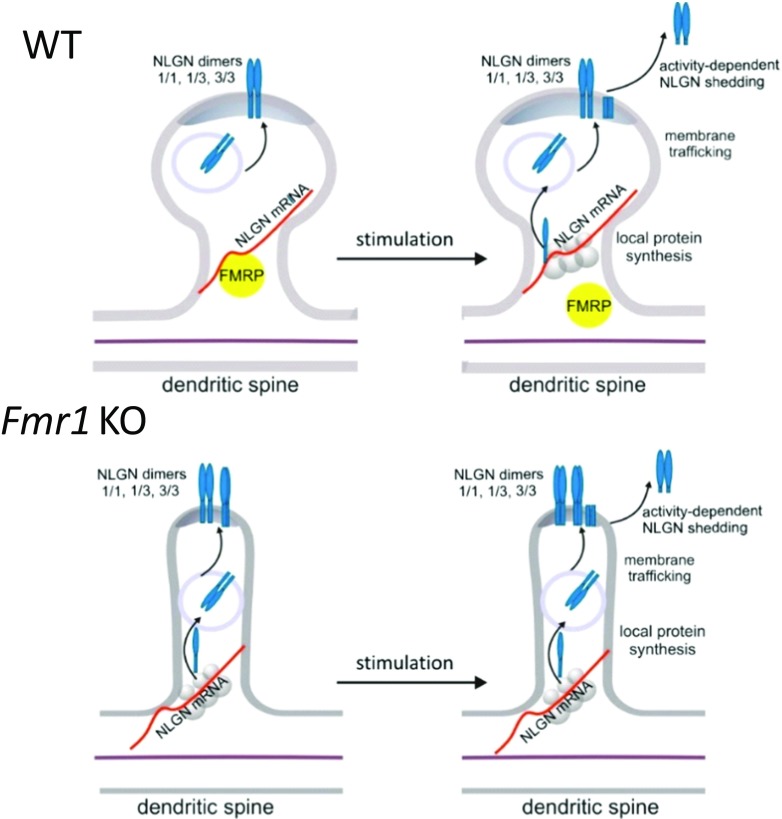
Schematic representation of activity-dependent NLGN1 and NLGN3 distribution at WT and *Fmr1* KO glutamatergic synapses. Model depicting NLGN1 and NLGN3 local protein synthesis, membrane trafficking and proteolytic cleavage in response to the stimulation at dendritic spines. The lack of FMRP in *Fmr1* KO mice leads to enhanced synaptic targeting of NLGN1 and NLGN3. Synaptic stimulation provokes very rapid proteolytic cleavage of NLGN1 and NLGN3 at both WT and *Fmr1* KO synapses

In the present study, we provide evidences that FMRP associates with *Nlgn1*, *Nlgn2*, and *Nlgn3* mRNAs and we confirm local translation of *Nlgn1*, *Nlgn2*, and *Nlgn3* mRNAs to be synaptically regulated by FMRP. We also describe enhanced targeting of NLGN1 and NLGN3 to the synapse of *Fmr1* KO mice. Using the in situ cross-linking method, we observed dynamic states of NLGNs turnover in synaptoneurosomes, which were the result of protein shuttling to the postsynaptic membrane and activity-induced rapid proteolytic cleavage of NLGN1, NLGN2, and NLGN3 in both WT and *Fmr1* KO synaptoneurosomes (Fig. 8).

Several lines of evidence reported the interaction of *Nlgns* transcripts with FMRP in the mouse brain [29, 46]. However, no direct association of FMRP with *Nlgns* mRNAs specifically at the synapse and in response to stimulation has been studied before. Here, we confirmed the presence of *Nlgn1*, *Nlgn2*, and *Nlgn3* mRNAs in the complex with FMRP using RNA co-IP with anti-FMRP antibody in synaptoneurosomes. These findings were further supported by colocalization studies of *Nlgn1*, *Nlgn2*, and *Nlgn3* mRNAs with FMRP in dendrites.

In the previous studies using high-throughput sequencing and proteomic methods, the large number of FMRP targets was identified in the mouse brain [29, 54]. Among them, one can distinguish many key synapse organizers such as neuroligins, but also neurexins, protocadherins, cadherins, ncams (neural cell adhesion molecule), contactins, or shank proteins. Altogether, it seems that FMRP controls the translation of the whole group of synapse organizing proteins, including neuroligins, which can be responsible for synapse abnormalities observed in FXS [55].

FMRP regulates the translation by ribosomal stalling on the target transcripts [29]. We observed that stimulation of NMDARs on synaptoneurosomes leads to significantly enhanced association of *Nlgns* mRNAs with translating polyribosomes. In the case of *Fmr1* KO mice, the fraction of *Nlgns* mRNA cosedimenting with the polysomes does not change upon the stimulation. This result is consistent with the previous studies showing other FMRP-dependent transcripts to behave similarly [36, 40, 49].

The results of our study on synaptoneurosomes and cultured neurons show elevated levels of synaptic NLGN1 and NLGN3 in *Fmr1* KO mice in basal conditions, which is yet unknown molecular phenotype of FXS. We postulate that described mechanism may be correlative to impairments observed in FXS. So far, the level of NLGNs was assessed only in total extracts from different brain regions and shown to be decreased in hippocampus and cerebellum for NLGN1, whereas no changes in NLGN2 and NLGN3 levels were detected in *Fmr1* KO mice [46]. However, the upregulated NLGNs level was detected in another genetic model of translational dysregulation: *4E-BP2* (eukaryotic initiation factor 4E-binding protein 2) KO mice, in which the authors observed enhanced eIF4E-dependent translation of *Nlgns* mRNAs in synaptosomal fractions [56]**.** Recently, it was also shown that the expression of NLGN1 is regulated by translation initiation by 4E-BP1 at the spinal cord synapses [57]. Thus, the synthesis of NLGNs is regulated post-transcriptionally at the synapse, suggesting the importance of its tight control.

Although we demonstrate that FMRP regulates synaptic translation of *Nlgn1*, *Nlgn2*, and *Nlgn3* mRNAs, we did not detect the elevated level of NLGN2 protein in *Fmr1* KO synaptoneurosomes. This may be attributed to the fact that we assessed translational regulation of *Nlgn1*, *Nlgn2*, and *Nlgn3* on mRNA level using very sensitive PCR-based methods (mRNA co-IP and polyribosome profiling). In the case of protein detection, we applied western blotting which is less sensitive method. This variance may also result from the age difference in the mice used for the polyribosome profiling and quantification of NLGN2 levels by western blotting. This discrepancy can be also related to the specific expression of NLGN2 on inhibitory synapses which are less abundant on the rodent cortical and hippocampal neurons [58–60]. We have shown that our synaptoneurosomal preparations contain both excitatory and inhibitory synapses and that during the preparation procedure, they are similarly enriched. The fraction of inhibitory synapses containing NLGN2 in synaptoneurosomes can be too small to detect quantitatively the differences in its expression between WT and *Fmr1* KO. One may not exclude the possibility that NLGN2 is specifically or more rapidly degraded than NLGN1 and NLGN3; however, little is known about regulation of protein degradation at the synapse.

One very characteristic neuroanatomical feature associated with FXS is altered dendritic spine structure, which appear abnormally long, thin, and filopodia-like, reminiscent of the immature spine precursors [61, 62]. Since the size of the excitatory postsynaptic contact is related to the dimension of dendritic spine, it is considered that thinner dendritic spine characteristic for FXS incorporate fewer receptors [63]. Surprisingly, we discovered that *Fmr1* KO incorporate more NLGN1 and NLGN3 into the postsynaptic membrane. We assume that this effect does not result from the increased spine and synapse number since the spine density does not differ between WT and *Fmr1* KO mice [61, 64, 65].

NLGN1-overexpressing mice show significant deficits in memory acquisition, altered dendritic spine morphology, and higher excitation to inhibition ratio in the hippocampus [66]. On the other hand, some of ASD-related mutations in *NLGNs* genes lead to intracellular retention of neuroligins [18–21]. Additionally, *Nlgn1* KO mice exhibit impairments in spatial learning and memory, hippocampal LTP and NMDA/AMPA ratio at cortico-striatal synapses [67]. This indicates that a precise and tightly regulated level of neuroligins at the synapse is important for providing synaptic integrity, probably due to their role in the recruitment of glutamate receptors at excitatory postsynapse [19, 25, 68]. In this context, our discovery of enhanced NLGN1 and NLGN3 level in *Fmr1* KO synapses adds to the molecular mechanisms contributing to the FXS phenotype. However, the reason for the enhanced NLGN1 and NLGN3 synaptic incorporation in *Fmr1* KO mice can also lie in dysregulated expression of proteins controlling NLGNs synaptic turnover.

Neuroligins function at the postsynaptic membrane as dimers, where they are delivered by a vesicular transport [15]. To distinguish between the surface and intracellular pool of neuroligins, we used in vitro proteins cross-linking with BS^3^ reagent. We detected a significantly higher abundance of surface NLGN1 and NLGN3 at *Fmr1* KO synapses in the basal, unstimulated synaptoneurosomes, and hippocampal cultures suggesting its increased membrane trafficking and incorporation into the postsynaptic density of *Fmr1* KO synapses. The time course stimulation of synaptoneurosomes, followed by cross-linking of extracellular proteins allowed us to show the reduction in the level of surface NLGN1-, NLGN2-, and NLGN3-containing dimers, due to their rapid activity-induced proteolytic cleavage. Concurrently, the intracellular pool of neuroligins was restored possibly due to the activation of their synaptic translation, followed by trafficking to the postsynaptic membrane. We hypothesize that this dynamic process is necessary to maintain synaptic boundaries and enable the structural plasticity of dendritic spines.

In the cross-linking experiments and in western blots on total synaptoneurosomes extracts, we consequently detected the decrease in total level of membrane-bound NLGN1, NLGN2, and NLGN3 that was accompanied by appearance of lower molecular weight bands representing C-terminal fragments of cleaved NLGN1, NLGN2, and NLGN3. We observed for the first time rapid and intense cleavage of NLGN1, NLGN2, and NLGN3 in both WT and *Fmr1* KO synaptoneurosomes, which was detectable as early as 2.5 min after NMDAR stimulation, corresponding to decline in their full-length protein level (at 120 kDa). The cleavage was equally efficient in WT and *Fmr1* KO synaptoneurosomes suggesting that this aspect of synaptic plasticity is not fully impaired at *Fmr1* KO synapses. However, we reckon that more detailed analysis is required to understand the molecular mechanism of activity-dependent NLGNs shedding at the synapse and its consequences for synaptic functions. To date, two specific secretory proteases were proposed to cleave NLGN1: matrix metalloproteinase 9 (MMP-9) [52] and disintegrin and metalloproteinase domain-containing protein 10 (ADAM-10) [53, 69–71]. Recently, the cleaved NLGN3 was detected in the medium from optogenetically stimulated acute cortical slices and reported to serve as the mitogen promoting glioma growth [70, 71]. NLGN1 and NLGN3 cleavage was also suggested by the proteomics study [69] and there is a report regarding NLGN2 cleavage in *Drosophila* [72].

In aggregate, our data provide new important insights into the molecular regulation of NLGNs at the synapse and the mechanism by which lack of FMRP might contribute to autism phenotypes. This may indicate new strategies for therapeutic advances.

## Electronic Supplementary Material

Fig. S1 Fluorescence in situ hybridization with control sense riboprobes. **a-c** FISH with sense riboprobes for **a** *Nlgn1*, **b** *Nlgn2* and **c** *Nlgn3* mRNA and immunofluorescence staining for FMRP were performed on DIV21 rat hippocampal neurons as negative control of FISH-IF experiment from Fig. 1g-i. Exposure times and image processing were identical for each sample as for antisense probes imaging. Scale bars, 5 μm. (PNG 201 kb)

High Resolution (EPS 1349 kb)

Fig. S2 The level of *Nlgn1*, *Nlgn2* and *Nlgn3* mRNAs is not changed in *Fmr1* KO synaptoneurosomes. *Nlgn1*, *Nlgn2*, *Nlgn3* mRNAs level in synaptoneurosomes isolated from WT and *Fmr1* KO mice assessed by qRT-PCR. Data are presented as mean values normalized to *Gapdh* mRNA level in synaptoneurosomes ± SEM, *n* = 6 mice/genotype. (PNG 46 kb)

High Resolution (EPS 1139 kb)

Fig. S3 Protein loading control. **a-c** TGX acrylamide gels visualization by Bio-Rad Gel Doc XR+ to confirm equal protein loading on the gels for subsequent immunodetection of **a** NLGN1, **b** NLGN2 **c** NLGN3 protein level in synaptoneurosomes shown in Fig. 3a-c. **d** Gel visualization and **e** subsequent immunodetection and quantification of β3-tubulin protein level in synaptoneurosomes isolated from WT and *Fmr1* KO mice which served as additional control of equal protein loading. Data are presented as mean values ± SEM, *n* = 5 mice/genotype. **f-h** TGX acrylamide gel visualization by Bio-Rad Gel Doc XR+ to confirm equal amount of protein was loaded onto the gels for subsequent immunodetection of **f** NLGN1, **g** NLGN2 **h** NLGN3 protein in DIV21 hippocampal cultures shown in Fig. 3d-f. **i** Gel visualization and **j** subsequent immunodetection and quantification of β3-tubulin protein level in DIV21 hippocampal cultures set from WT and *Fmr1* KO mice which served as additional control of equal protein loading. Data are presented as mean values ± SEM, *n* = 6. (PNG 594 kb)

High Resolution (EPS 4396 kb)

Fig. S4 Protein loading control. **a-g** TGX acrylamide gel visualization by Bio-Rad Gel Doc XR+ to confirm equal amount of protein was loaded onto the gels for subsequent immunodetection of activity-dependent cleavage of **a**, **d** NLGN1, **b**, **e** NLGN2 **c**, **f** NLGN3 protein in WT and *Fmr1* KO synaptoneurosomes shown in Fig. 7a-f and **g** NLGN3 protein in WT and *Nlgn3* KO synaptoneurosomes shown in Fig. 7g. (PNG 435 kb)

High Resolution (EPS 3256 kb)
