# Supplementary figures and images for: Neuroligin 1, 2, and 3 Regulation at the Synapse: FMRP-Dependent Translation and Activity-Induced Proteolytic Cleavage
Source: Mol Neurobiol. 2018 Jul 28;56(4):2741–59. doi: 10.1007/s12035-018-1243-1 (PMC6459971; doi:10.1007/s12035-018-1243-1)

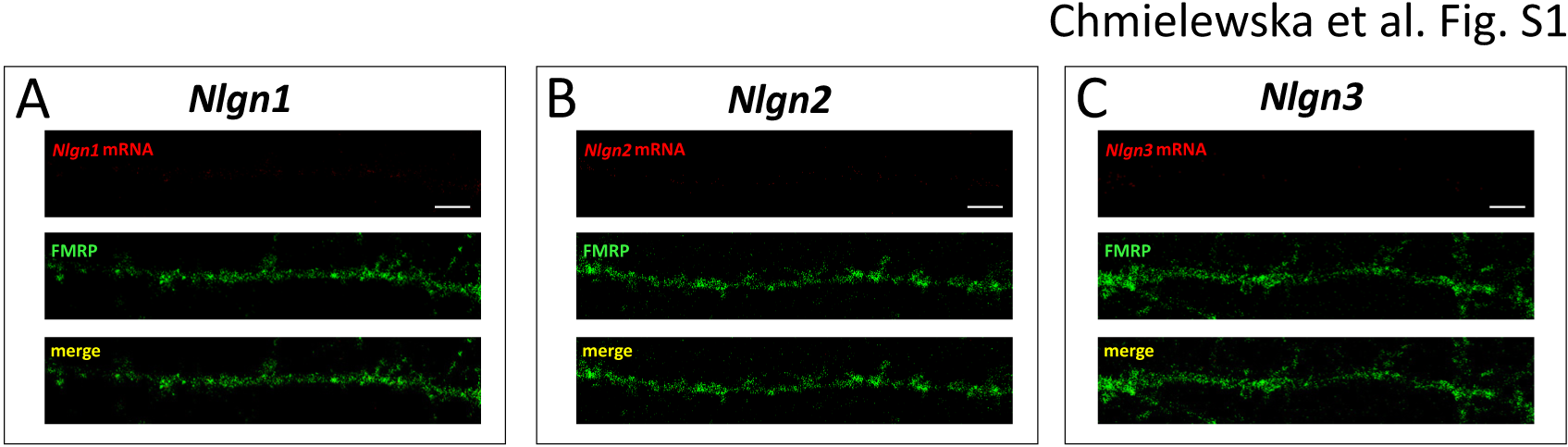

Supplement: Supplementary file 1 — Fluorescence in situ hybridization with control sense riboprobes. a-c FISH with sense riboprobes for a Nlgn1, b Nlgn2 and c Nlgn3 mRNA and immunofluorescence staining for FMRP were performed on DIV21 rat hippocampal neurons as negative control of FISH-IF experiment from Fig. 1g-i. Exposure times and image processing were identical for each sample as for antisense probes imaging. Scale bars, 5 μm. (PNG 201 kb) [file 12035_2018_1243_Fig9_ESM.png]

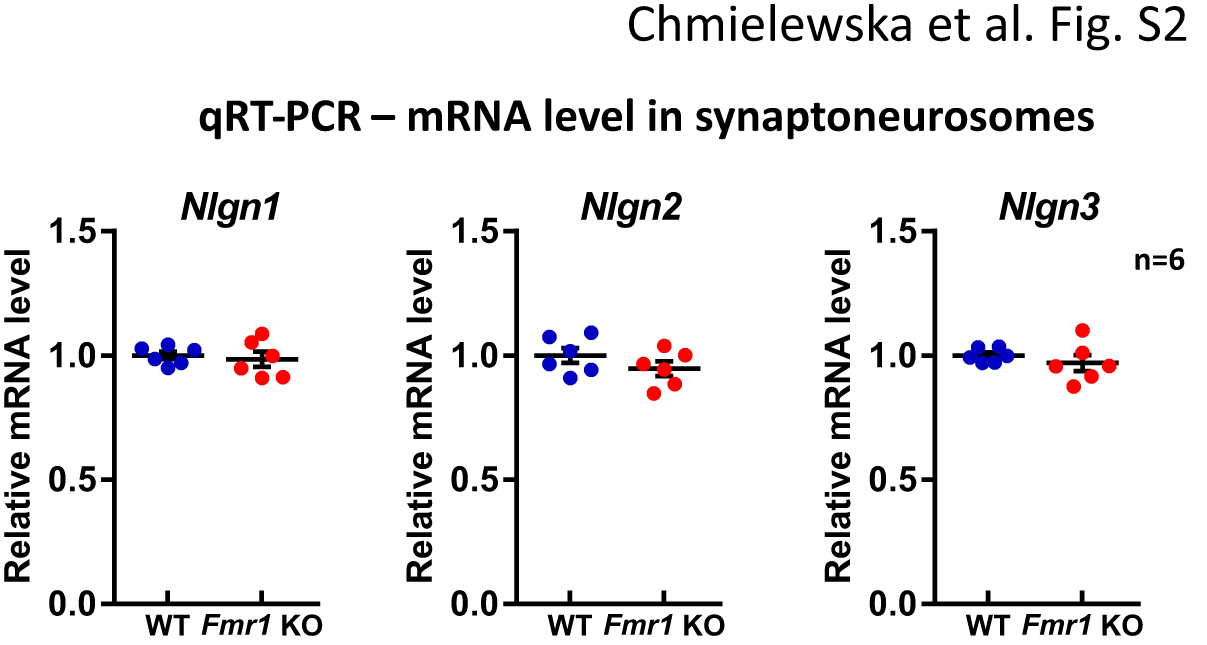

Supplement: Supplementary file 3 — The level of Nlgn1, Nlgn2 and Nlgn3 mRNAs is not changed in Fmr1 KO synaptoneurosomes. Nlgn1, Nlgn2, Nlgn3 mRNAs level in synaptoneurosomes isolated from WT and Fmr1 KO mice assessed by qRT-PCR. Data are presented as mean values normalized to Gapdh mRNA level in synaptoneurosomes ± SEM, n = 6 mice/genotype. (PNG 46 kb) [file 12035_2018_1243_Fig10_ESM.png]

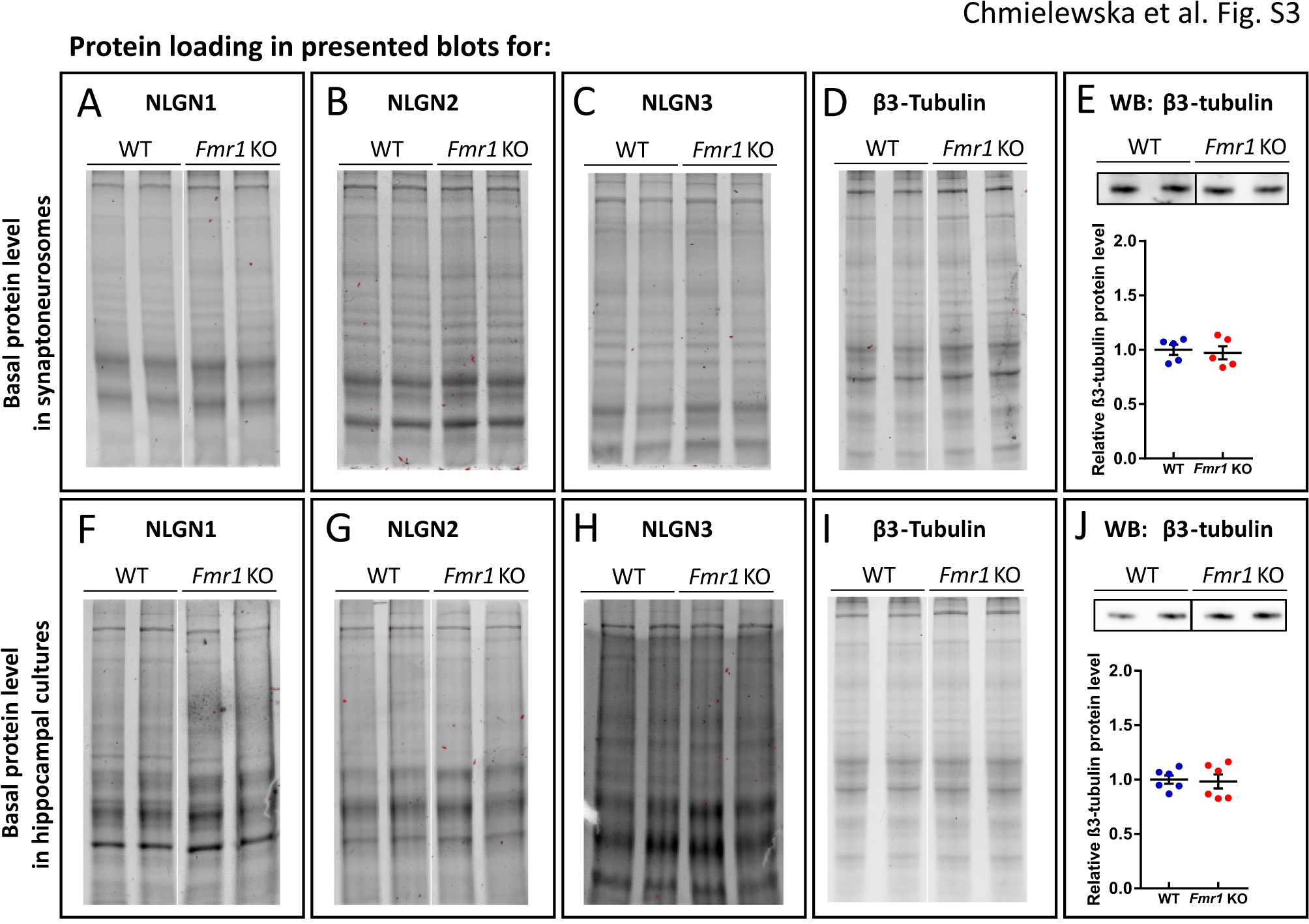

Supplement: Supplementary file 5 — Protein loading control. a-c TGX acrylamide gels visualization by Bio-Rad Gel Doc XR+ to confirm equal protein loading on the gels for subsequent immunodetection of a NLGN1, b NLGN2 c NLGN3 protein level in synaptoneurosomes shown in Fig. 3a-c. d Gel visualization and e subsequent immunodetection and quantification of β3-tubulin protein level in synaptoneurosomes isolated from WT and Fmr1 KO mice which served as additional control of equal protein loading. Data are presented as mean values ± SEM, n = 5 mice/genotype. f-h TGX acrylamide gel visualization by Bio-Rad Gel Doc XR+ to confirm equal amount of protein was loaded onto the gels for subsequent immunodetection of f NLGN1, g NLGN2 h NLGN3 protein in DIV21 hippocampal cultures shown in Fig. 3d-f. i Gel visualization and j subsequent immunodetection and quantification of β3-tubulin protein level in DIV21 hippocampal cultures set from WT and Fmr1 KO mice which served as additional control of equal protein loading. Data are presented as mean values ± SEM, n = 6. (PNG 594 kb) [file 12035_2018_1243_Fig11_ESM.png]

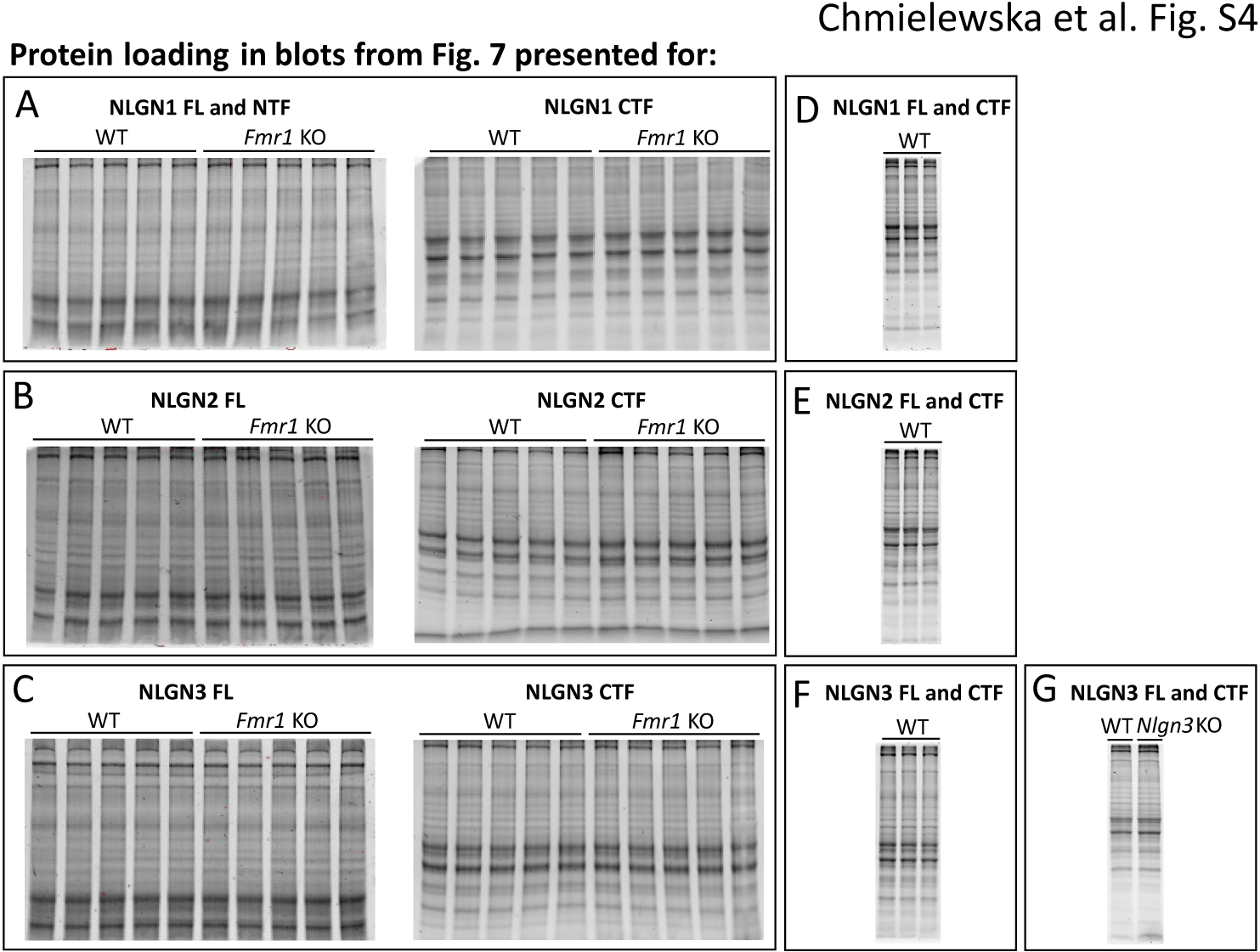

Supplement: Supplementary file 7 — Protein loading control. a-g TGX acrylamide gel visualization by Bio-Rad Gel Doc XR+ to confirm equal amount of protein was loaded onto the gels for subsequent immunodetection of activity-dependent cleavage of a, d NLGN1, b, e NLGN2 c, f NLGN3 protein in WT and Fmr1 KO synaptoneurosomes shown in Fig. 7a-f and g NLGN3 protein in WT and Nlgn3 KO synaptoneurosomes shown in Fig. 7g. (PNG 435 kb) [file 12035_2018_1243_Fig12_ESM.png]
